# Mental health in the COVID-19 pandemic: A longitudinal analysis of the CLoCk cohort study

**DOI:** 10.1371/journal.pmed.1004315

**Published:** 2024-01-24

**Authors:** Laura Panagi, Simon R. White, Snehal M. Pinto Pereira, Manjula D. Nugawela, Isobel Heyman, Kishan Sharma, Terence Stephenson, Trudie Chalder, Natalia K. Rojas, Emma Dalrymple, Kelsey McOwat, Ruth Simmons, Olivia Swann, CLoCk Consortium, Tamsin Ford, Roz Shafran

**Affiliations:** 1 Department of Psychiatry, University of Cambridge, Cambridge, United Kingdom; 2 Division of Surgery & Interventional Science, Faculty of Medical Sciences, University College London, London, United Kingdom; 3 UCL Great Ormond Street Institute of Child Health, University College London, London, United Kingdom; 4 Division of Neuroscience & Experimental Psychology, University of Manchester, Manchester, United Kingdom; 5 Department of Psychological Medicine, Institute of Psychiatry, Psychology and Neuroscience, King’s College London, London, United Kingdom; 6 Immunisation Department, Public Health England, London, United Kingdom; 7 Centre for Medical Informatics, Usher Institute, University of Edinburgh, Edinburgh, United Kingdom; Harvard Medical School, UNITED STATES

## Abstract

**Background:**

Little is known about the long-term mental health consequences of the pandemic in children and young people (CYP), despite extremely high levels of exposure to the Severe Acute Respiratory Syndrome Coronavirus 2 (SARS-CoV-2) virus and the disruption to schooling and leisure activities due to the resultant restrictions. There are mixed findings from systematic reviews of how the pandemic affected CYP’s mental health, which may be due to heterogeneous methods and poor quality studies. Most, but not all, suggest deterioration in mental health but population level studies may obscure the differing experiences of subgroups. The study questions are: (i) are there subgroups of CYP with distinct mental health profiles over the course of the second year of the Coronavirus Disease 2019 (COVID-19) pandemic (between April 2021 and May 2022); and (ii) do vulnerability factors influence CYP’s mental health trajectories.

**Methods and findings:**

A matched longitudinal cohort study of non-hospitalised test-positive and test-negative 11- to 17-year-old CYP in England were recruited from the UK Health Security Agency having undergone PCR testing for COVID-19. They completed the Strengths and Difficulties Questionnaire (SDQ) at least twice over a 12-month follow-up period. Overall, 8,518 of 17,918 (47.5%) CYP who returned their first SDQ at 3 or 6 months post-testing were included in the analytical sample. Associations between age, sex, ethnicity, socioeconomic status (SES), and an educational health and care plan (EHCP, indicating special educational needs) on SDQ score trajectories were examined separately, after adjusting for PCR test result. Findings from multilevel mixed-effects linear regression model showed that on average mental health symptoms as measured by the total SDQ score increased over time (*B* = 0.11 (per month), 95% CI = 0.09 to 0.12, *p* < 0.001) although this increase was small and not clinically significant. However, associations with time varied by age, such that older participants reported greater deterioration in mental health over time (*B* = 0.12 (per month), 95% CI = 0.10 to 0.14 for 15 to 17y; 0.08 (95% CI = 0.06 to 0.10) for 11 to 14y; p_interaction_ = 0.002) and by sex, with greater deterioration in girls. Children with an EHCP experienced less deterioration in their mental health compared to those without an EHCP. There was no evidence of differences in rate of change in total SDQ by ethnicity, SES, or physical health. Those with worse prior mental health did not appear to be disproportionately negatively affected over time. There are several limitations of the methodology including relatively low response rates in CLoCk and potential for recall bias.

**Conclusions:**

Overall, there was a statistically but not clinically significant decline in mental health during the pandemic. Sex, age, and EHCP status were important vulnerability factors that were associated with the rate of mental health decline, whereas ethnicity, SES, and prior poor physical health were not. The research highlights individual factors that could identify groups of CYP vulnerable to worsening mental health.

## Abstract 2

Laura Panagi and co-workers study mental health among children and young people in England during the COVID-19 pandemic.

## Author summary

**Why was this study done?:**

- Almost all children and young people (CYP) have been exposed to Coronavirus Disease 2019 (COVID-19) and lived through a pandemic. The pandemic appears to have had negative implications for CYP’s mental health across the world although the quality of research is often poor.
- The large-scale CYP with Long COVID (CLoCk) study conducted in England allows researchers to describe the natural course of mental health symptoms at 3, 6, and 12 months after testing using a robust measure of emotional and behavioural difficulties (the Strengths and Difficulties Questionnaire (SDQ)).
- The CloCk study allows exploration of what factors are associated with developing or worsening of mental health problems.
- We wanted to know whether there are subgroups of CYP with distinct mental health characteristics and to understand how specific vulnerability factors are associated with CYP’s mental health over the pandemic.

**What did the researchers do and find?:**

- We asked CYP who had tested positive or negative for COVID-19 in England between September 2020 and March 2021 to complete a questionnaire about their mental health 3, 6, and/or 12 months after testing. The study uses information from 8,518 CYP who agreed to take part and filled in at least 2 questionnaires.
- We did not find subgroups of CYP with distinct mental health profiles over the course of the second year of the COVID-19 pandemic, but found that although there was a statistical deterioration in mental health over time, overall, the mean deterioration was relatively small and not clinically meaningful.
- We found that age, sex, and whether a CYP had an educational health and care plan (EHCP, which is typically given when there are learning difficulties) were all associated with the rate of change in mental health over time.
- Older teenagers and girls had on average a greater decline in their mental health than younger CYP and boys. In contrast, CYP with an EHCP experienced a smaller decline in their mental health during the pandemic, although overall experienced much higher levels of difficulties compared to their non-EHCP counterparts.

**What do these findings mean?:**

- Changes in the mental health of CYP during the pandemic is somewhat unclear but our findings indicate that there were differences in behavioural, emotional, and attentional difficulties in children with special educational needs compared to the rest of the sample.
- Our findings suggest that CYP in their mid to late teens are particularly vulnerable and continue to struggle with their mental health. However, it is important to consider that within the findings there were a lot of differences between individuals.
- Our findings suggest that on average rates of change in mental health during the pandemic differed by age, sex, and whether the CYP had an EHCP.
- We need long-term follow-up of such cohorts to further understand the mental health trajectories of young people as we emerge from the pandemic.

## 1 Introduction

Severe Acute Respiratory Syndrome Coronavirus 2 (SARS-CoV-2) infection in children and young people (CYP) has generally been asymptomatic or a mild illness compared to adults [1] although the scale of infection is unprecedented, with 90% of CYP in countries such as the United Kingdom exposed to the virus [2] and all living through a global pandemic. Increasing evidence suggests that the pandemic has had an adverse impact on CYP’s mental health [3,4]. Of the studies specifically investigating mental health symptoms in CYP, one of the first was the UK-based Co-SPACE study which reported on a convenience sample of 3,322 children and adolescents (aged 4 to 16 years) for whom parents completed a baseline survey and at least 1 follow-up survey between March 2020 and May 2021. Most participants followed low stable or moderate stable symptom trajectories, with 14% to 31% experiencing very high, high stable, or increasing mental health difficulties [5]. A recent follow-up report of the Co-SPACE study provided an overview of data with 1,977 participants completing the 24-month follow-up survey and concluded that CYP’s behavioural and attentional difficulties remained relatively stable since July 2021 but were elevated for children with special educational needs and for those from low-income families [6].

A systematic review of the mental health changes among over 96,000 CYP before and during the Coronavirus Disease 2019 (COVID-19) pandemic [7] identified 21 studies from 11 countries published between January 2020 and March 2021 and found that most studies reported a deterioration in the mental health of CYP over time, with increased depression, anxiety, loneliness, and psychological distress after the pandemic started. Another systematic review team’s search extended until February 2022, retrieved 51 studies, and reported mixed evidence of impact on some measures of CYP’s mental health, with many poor quality and heterogeneous methods that restricted meta-analysis [8]. Both review teams concluded that there is a very limited number of large-scale, repeated cross-sectional or longitudinal studies with clear sampling frames. One exception to this is the large-scale Children and Young People with Long COVID (CLoCk) study [9] which includes standardised measures of mental health during the pandemic. It is the largest national, matched longitudinal cohort study of long COVID in CYP in England. Non-hospitalised CYP report post-COVID health symptoms after a laboratory-confirmed SARS-CoV-2 infection and are compared to age-, sex-, and geographically-matched CYP with a laboratory-confirmed SARS-CoV-2 negative test [9,10]. The study sampled from all CYP who had a PCR test between September 2020 and March 2021. The design of the study allows us to describe the natural course of mental health symptoms at 3, 6, and 12 months after testing using a standardised measure of emotional and behavioural symptoms. It also allows for exploration of vulnerability factors that might be associated with the symptom trajectory.

Previous findings from the CLoCk study revealed little difference in the prevalence of total mental health difficulties between the test-positive and test-negative CYP using a subsample of 1,808 CYP with full data at 3, 6, and 12 months post-testing [11]. However, the report did not include an analysis of vulnerability factors to mental health difficulties, although vulnerability factors such as sex, age, ethnicity, physical and mental health predicted long COVID 3 months after testing [12]. Furthermore, in adults, a national, probability-based sample, found that those with poorer mental health during the pandemic were more likely to have preexisting mental or physical ill-health, to live in deprived neighbourhoods, and be of Asian, black, or mixed ethnicity [13].

The current report focuses on participants in the CLoCk study who completed validated mental health measures at least twice over the 12-month follow-up period. The objectives of this study are to (i) determine whether there are subgroups of CYP with distinct mental health profiles over the course of the second year of the COVID-19 pandemic (between April 2021 and May 2022); and (ii) understand which vulnerability factors are associated with CYP’s mental health profiles. Based on previous literature (e.g., [6,13]), it was hypothesised that girls, those with special educational needs, from low-income families, and with poorer pre-pandemic physical and mental health would have a worse mental health profiles than young people without these vulnerability factors.

## 2 Methods

### 2.1 Participants and procedure

The CLoCk study is a cohort study of SARS-CoV-2 PCR-positive CYP aged 11 to 17 years, matched at study invitation, on month of testing, age, sex, and geographical area to SARS-CoV-2 PCR-negative CYP using the national SARS-CoV-2 testing dataset held by the United Kingdom Health Security Agency. Details of the study design are reported elsewhere [9].

The CLoCk study collects questionnaire data on >30,000 CYP up to 24 months after a SARS-CoV-2 PCR test, taken between September 2020 and March 2021 [9]. CYP tested between October and December 2020 provided their first questionnaire data 6 months after testing and were approached again at 12 months post-testing to complete the same questionnaire (a subset who tested in December 2020 provided their first questionnaire data 12 months after testing). CYP tested between January and March 2021 first provided data 3 months post-testing and were approached again at 6 and 12 months post-testing to complete the same questionnaire. Since 24-month data are not yet available for analysis, we examined data up to the 12-month follow-up. CYP tested in September 2020 and the December 2020 subset were not included in the analytical sample as they provided data at only once (12 months post-testing). At first invitation 3 or 6 months post-testing, 17,918 participants filled in their first questionnaire (which included reporting on their current mental health symptoms). We included participants who filled in the questionnaire at least twice over the follow-up period (excluded *n* = 9,400). The final analytic sample consisted of 8,518 CYP. All participants gave written informed consent to take part in the study.

### 2.2 Study measures

We collected information from the national SARS-CoV-2 testing dataset held by the United Kingdom Health Security Agency on SARS-CoV-2 PCR result (negative, positive); age at time of testing (examined as 11 to 14 versus 15 to 17, based on education key stage groups in England); sex (girls, boys), and Index of Multiple Deprivation (IMD, derived from CYP’s lower super output area (a small local area level-based geographical hierarchy) as a proxy of socioeconomic status, grouped from most [quintile 1] to least [quintile 5] deprived).

The online questionnaires were designed with the International Severe Acute Respiratory and emerging Infection Consortium Paediatric Working Group. A carer could assist participants who requested it and those with special educational needs or disabilities. In the first online questionnaire, participants reported their ethnicity (Asian/Asian British, Black/African/Caribbean, Mixed, Other, Unknown/Prefer not to say, White) and whether before the COVID-19 pandemic in early March 2020, they had an Education Health and Care Plan (EHCP) indicating a need for extra support for learning at school (yes, no). Additionally, participants rated their mental and physical health before testing using the questions “How was your mental/physical health in general before your COVID-19 test?” (5 categories ranging from very good to very poor).

In all questionnaires, participants filled in the globally recognised measure of mental health difficulties in CYP, the Strengths and Difficulties Questionnaire (SDQ) [14]. The SDQ, which is widely used in epidemiological research, is comprised of 25 items enquiring about emotional and behavioural attributes of the young person which are combined to form 5 subscales. The emotional symptoms subscale includes items that ask about fears, worries, misery, nerves, and somatic symptoms. The conduct problems subscale contains items about anger outbursts, obedience, fighting, lying, and stealing. The hyperactivity subscale covers restlessness, fidgeting, concentration, distractibility, and impulsivity. The peer relationships subscale enquires about popularity, victimisation, isolation, friendship, and ability to relate to children as compared to adults, and the prosocial skills subscale covers consideration of others, ability to share, kindness to younger children, helpfulness when other children are distressed, and willingness to volunteer to comfort. Subscales scores range from 0 to 10, with higher scores indicating greater difficulties, except the prosocial skills subscale where elevated scores reflect greater skills. All the subscales except the prosocial skills subscale are summed to produce a total difficulties score ranging from 0 to 40, with an elevated score reflecting poorer mental health. CYP tested between October and December 2020 were asked to complete the SDQ twice, at 6 and 12 months post-testing. CYP tested between January and March 2021 were asked to complete the online SDQ thrice, at 3, 6, and 12 months post-testing.

### 2.3 Statistical analysis

After discussing options with all coauthors, LP, SRW, and SMPP finalised the analytic plan in October 2022, which was revised in November 2022 (see explanation below). Further analyses were added in February 2023 in response to reviewer comments (addition of associations of PCR status with mental health trajectories).

All analyses were performed in STATA v.17.0. First, we described the sample at the different data collection schedules (Table 1). Means (M) and standard deviations (SDs) of the SDQ subscales for the data collection sweeps are also presented (S1 Table). Those included in the analytic sample (participants with at least 2 SDQ measurements) were compared to those excluded, on the vulnerability factors of interest, using Chi-square tests (S2 Table).

**Table 1:** Sample characteristics by data collection schedule: mean (standard deviation)/*N*(%).

Variable	3m post-testing (*n* = 4,292)	6m post-testing (*n* = 8,076)	12m post-testing (*n* = 7,404)
**SDQ total score**	11.3 (6.4)	11.3 (6.5)	11.8 (6.6)
**SARS-CoV-2 PCR result** *			
Negative	11.2 (6.4)	11.5 (6.5)	12.0 (6.6)
Positive	11.4 (6.3)	11.2 (6.4)	11.7 (6.5)
Age* **(years)**			
11–14	10.1 (6.4)	10.3 (6.6)	10.7 (6.8)
15–17	12.1 (6.2)	12.1 (6.3)	12.6 (6.3)
**Sex** *			
Girl	12.0 (6.4)	12.1 (6.5)	12.7 (6.6)
Boy	10.0 (6.0)	9.8 (6.0)	10.3 (6.2)
**Ethnicity**			
Asian/Asian British	10.5 (6.1)	10.7 (6.1)	11.3 (6.3)
Black/African/Caribbean	10.4 (6.1)	10.9 (6.4)	11.6 (5.8)
Mixed	11.6 (6.3)	11.9 (6.2)	12.2 (6.5)
Other	10.9 (6.1)	10.9 (5.9)	11.3 (6.2)
Unknown/prefer not to say	12.6 (7.3)	12.8 (6.6)	13.8 (6.8)
White	11.5 (6.4)	11.4 (6.5)	11.9 (6.6)
**IMD** *			
1 –most deprived	11.9 (6.5)	12.1 (6.6)	12.7 (6.7)
2	11.7 (6.5)	11.6 (6.6)	12.3 (6.7)
3	11.5 (6.4)	11.5 (6.5)	12.0 (6.7)
4	10.8 (6.2)	10.9 (6.4)	11.3 (6.5)
5 –least deprived	10.6 (6.2)	10.7 (6.2)	11.2 (6.3)
**Prior mental health**			
Poor/very poor	18.8 (5.6)	18.5 (6.2)	18.4 (6.2)
Okay	13.7 (5.6)	14.0 (5.8)	14.4 (6.0)
Good/very good	8.9 (5.4)	9.0 (5.4)	9.7 (5.7)
**Prior physical health**			
Poor/very poor	16.3 (7.4)	16.4 (7.1)	16.8 (7.0)
Okay	14.2 (6.3)	14.2 (6.4)	14.4 (6.5)
Good/very good	10.3 (6.0)	10.5 (6.2)	11.1 (6.4)
**EHCP status**			
No	11.0 (6.2)	11.1 (6.3)	11.6 (6.5)
Yes	15.5 (7.0)	15.5 (7.2)	15.7 (7.2)

**Note.** Participants who tested between October and December 2020 were asked to complete the SDQ twice, at 6 and 12 months post-testing. Participants who tested between January and March 2021 were asked to complete the SDQ thrice, at 3, 6, and 12 months post-testing; not all participants took part at all 3 time points but each participant filled in the SDQ at least twice. Three months post-testing reflects the time window: 13 April 2021 to 17 October 2021; 6 months post-testing reflects the time window: 13 April 2021 to 17 February 2022; 12 months post-testing reflects the time window: 5 October 2021 to 10 May 2022.

*Ascertained from the UK Health Security Agency database.

EHCP, educational health and care plan; IMD, Index of Multiple Deprivation; m, months; PCR, polymerase chain reaction; SD, standard deviation; SDQ, Strengths and Difficulties Questionnaire.

We attempted to determine whether there were subgroups of CYP with distinct mental health profiles over the course of the second year of the COVID-19 pandemic (objective i) using latent class mixed-effects models, allowing for multiple classes of individuals with class-specific trajectories. However, based on the Bayesian information criterion (BIC), there was no evidence for multiple classes. Specifically, the BIC value was lower in the 1-class solution as compared with the models with 2 to 7 classes. Nevertheless, we observed substantial variability in SDQ scores over time. Hence, multilevel mixed-effects models with a random intercept were used to examine the mean trajectory of emotional and behavioural difficulties in CYP during the pandemic, using the continuous SDQ total difficulties score. Time was modelled as a continuous variable calculated as day of survey completion, centred at the first day of survey data collection (13 April 2021; the first day took the value of 0); days were then divided by 30 to aid interpretation on a month-metric. We considered several functions to accurately model the relationship between total difficulties and time including linear, quadratic, and cubic models. The linear model provided the best fit for the data. Next, to understand which vulnerability factors were associated with CYP’s mental health profiles (objective ii), 7 additional models were fitted by separately modelling the association of age, sex, ethnicity, IMD, EHCP status, prior mental health, and prior physical health on the start of the mental health trajectory (i.e., the intercept) and its rate of change over the year using an interaction term between the variable of interest and time (i.e., the slope). All models were adjusted for the CYP’s baseline SARS-CoV-2 PCR result. Finally, we also fitted an eighth model, exploring the association between baseline PCR status and subsequent mental health trajectories. Results from mixed-effects models are presented as beta coefficients on a per month scale, with standard error (SE), 95% confidence interval (CI), and *p* value (S3 Table) and as plots of the mean trajectories over time. This study is reported as per the Strengthening the Reporting of Observational Studies in Epidemiology (STROBE) guideline (see S1 STROBE Checklist).

### 2.4 Ethical approval and consent

Ethical approval was provided by the Yorkshire & The Humber—South Yorkshire Research Ethics Committee (REC reference: 21/YH/0060; IRAS project ID:293495). Public Health England (now UKHSA) has legal permission, provided by Regulation 3 of The Health Service (Control of Patient Information) Regulations 2002, to process patient confidential information for national surveillance of communicable diseases. Parents/carers were sent an invitation by post sent through PHE/UKHSA on behalf of the research team with a link to the website with the relevant information sheets and consent forms and they had the opportunity to ask any questions about the study. Parents/carers of CYP under 16 years of age were asked to complete an online parent/carer consent form. The young person was also asked to complete an online assent form to indicate their agreement. Consent was asked online from 16- to 17-year-olds (using the Young Person Consent Form) in line with Health Research Authority recommended processes.

## 3 Results

### 3.1 Sample characteristics

Overall, 8,518 of 17,918 (47.5%) CYP who returned their first SDQ at 3 or 6 months post-testing were included in the analytical sample. Older participants, girls, participants of white ethnicity, and those who were least deprived were more likely to be included in the analytic sample (S2 Table). Table 1 presents sample characteristics by data collection schedule (3, 6, and 12 months post-testing).

### 3.2 Mental health over time (presented as unit change per month)

**Fig 1:**
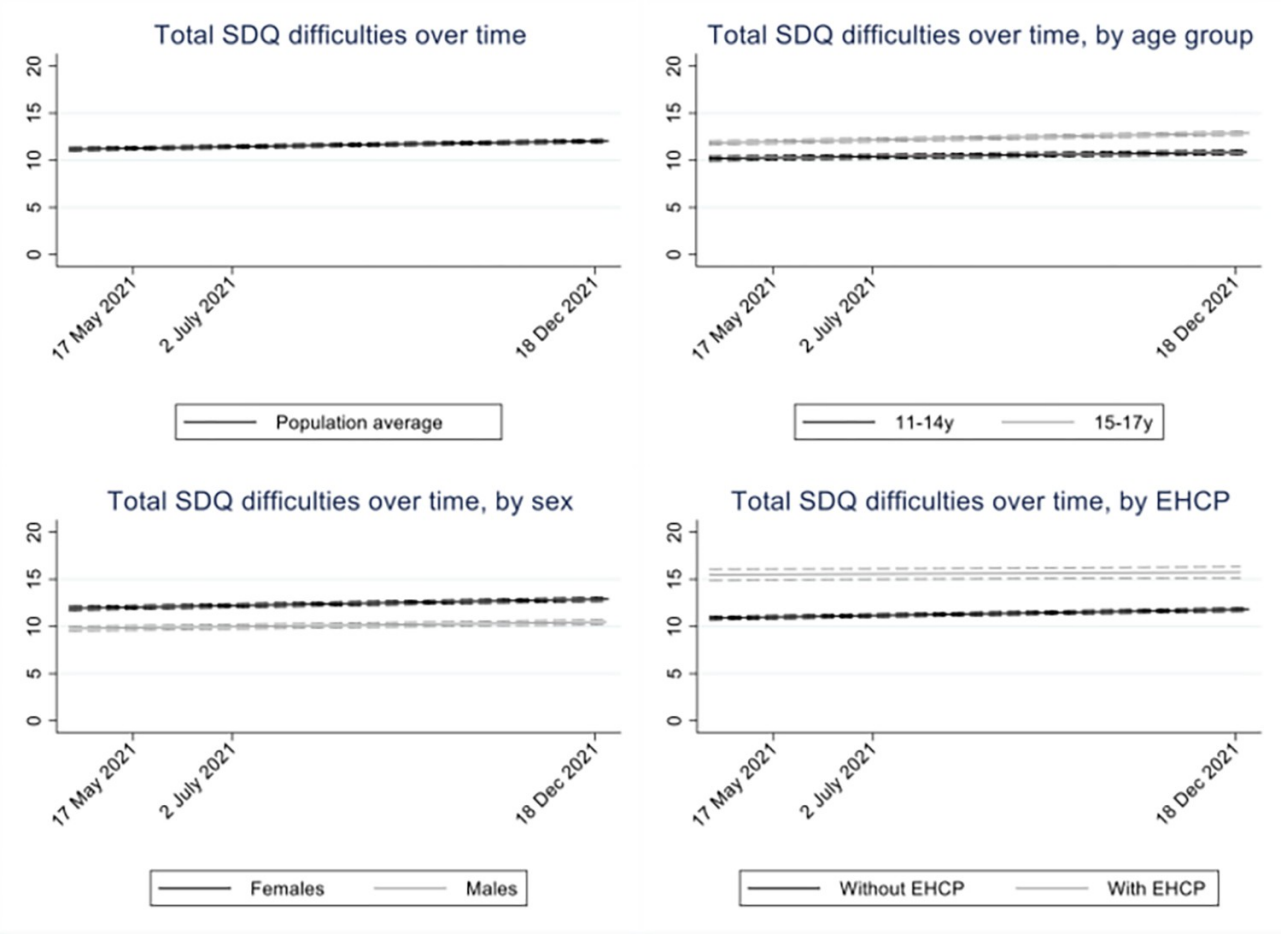
Average (95% CIs) total difficulties score (SDQ) over time (*N* = 8,518), shown from models for the whole population, by age, sex, and EHCP status. Footnote: Time was modelled as a continuous variable; dates reflect the mean questionnaire return time. All models adjusted for initial SARS-CoV-2 PCR result (i.e., trajectories in figure are for test-negatives). The intraclass correlations for the models was 0.77 (total population, by age group and EHCP) and 0.76 (by sex). CI, confidence interval; EHCP, educational health and care plan; SARS-CoV-2, Severe Acute Respiratory Syndrome Coronavirus 2; SDQ, Strengths and Difficulties Questionnaire.

On average there was a small increase in the total SDQ score over time (0.11 per month, 95% CI: 0.09 to 0.12; Fig 1 and S3 Table).

On average, the 15- to 17-year-olds had 1.65 (95% CI = 1.37 to 1.93) higher total SDQ score at the start of the trajectory compared to 11- to 14-year-olds. There was also a significant time by age-group (at baseline) interaction, suggesting that older participants showed a faster rate of increase on the SDQ over time (by 0.04 per month, 95% CI: 0.02 to 0.07; Fig 1 and S3 Table), compared to younger CYP.

On average, boys had lower baseline SDQ scores (−2.15, 95% CI: −2.44 to −1.86) compared to girls and showed a statistically significant, but numerically small, slower rate of increase on the SDQ over time (by −0.03 per month, 95% CI: −0.06 to −0.004), indicating faster deterioration in mental health over time for girls (Fig 1 and S3 Table).

While CYP with an EHCP had higher SDQ scores at the start of the trajectory on average (by 4.60, 95% CI: 3.99 to 5.21), they had a slower increase in scores over time (−0.08 per month, 95% CI: −1.14 to −0.02) compared to CYP without EHCP (Fig 1 and S3 Table).

Compared to CYP of Asian/Asian British ethnicity, CYP of mixed ethnicity (1.17, 95% CI: 0.42 to 1.92), those who preferred not to disclose their ethnicity (2.56, 95% CI: 0.42 to 4.70), and white participants (0.76, 95% CI: 0.35 to 1.18) had elevated baseline total difficulties scores. However, there was no significant difference in rate of change in SDQ scores by ethnicity (p_interaction_ = 0.96), indicating a similar rate of change in mental health for all ethnicities (S1 Fig and S3 Table).

Compared to the most socioeconomically deprived group, on average CYP from less deprived groups reported fewer initial emotional and behavioural difficulties (quintile 3 versus 1: −0.67, 95% CI: −1.14 to −0.21; quintile 4 versus 1: −1.19, 95% CI: −1.64 to −0.74; quintile 5 versus 1: −1.47, 95% CI: −1.91 to −1.04). There were no differences in the rate of change in SDQ scores by IMD (p_interaction_ = 0.82), suggesting the rate of change in SDQ was similar for all levels of deprivation (S2 Fig and S3 Table).

**Fig 2:**
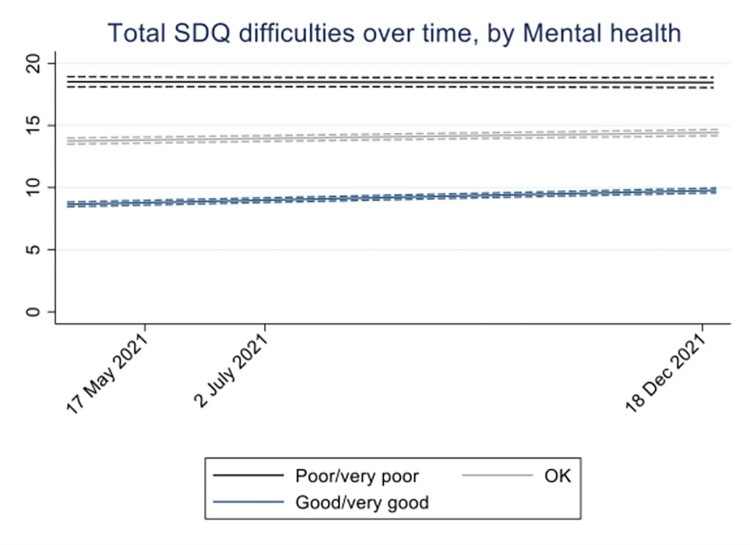
Average (95% CIs) total difficulties score (SDQ) over time by prior mental health (*N* = 8,518). Footnote: Time was modelled as a continuous variable; time points reflect the mean questionnaire return time (in months) within each time window. The model was adjusted for SARS-CoV-2 PCR result. The individual-level random effect and residual variance are 22.5 and 9.56, respectively, giving an intraclass correlation of 0.70. CI, confidence interval; SARS-CoV-2, Severe Acute Respiratory Syndrome Coronavirus 2; SDQ, Strengths and Difficulties Questionnaire.

As expected, CYP with better prior mental health (versus poor/very poor mental health) had lower total difficulties scores at the start of the trajectory (Okay: −4.78, 95% CI: −5.25 to −4.32; good/very good: −9.89, 95% CI: −10.32 to −9.45) but those with better prior mental health showed greater increases in scores over follow-up (p_interaction_ < 0.001, Okay: 0.09 per month, 95% CI: 0.04 to 0.14; good/very good: 0.14 per month, 95% CI: 0.10 to 0.19); suggesting that CYP with better prior mental health experienced a faster worsening during the pandemic (Fig 2 and S3 Table).

CYP with better prior physical health (versus poor/very poor physical health) had lower total difficulties scores at the start of the trajectory (Okay: −2.39, 95% CI: −3.40 to −1.39; good/very good: *B* = −6.14, 95% CI: −7.12 to −5.16), and there were no differences in the rate of change in scores over time (p_interaction_ = 0.10, S3 Fig and S3 Table).

Finally, there was no evidence of difference in the start of the trajectory or change over time by baseline PCR status (S4 Fig and S3 Table).

## 4 Discussion

The first aim of this study was to determine the course of mental health difficulties in CYP during the second year of the COVID-19 pandemic using a large, national cohort of participants, recruited using a public health approach. The data indicated that although there was a statistical deterioration in mental health over time, overall, the average deterioration across the population was relatively small and not clinically meaningful. Furthermore, there were not distinct subgroups within the population, although other groups have reported this from studies conducted earlier [5,13]. The second aim was to understand associations between vulnerability factors and young people’s mental health over this time period, and we found important associations with age, sex, and EHCP status. Older participants experienced greater deterioration in their mental health symptoms over time compared to younger CYP, and as did girls compared to boys. In contrast, CYP with an EHCP experienced a smaller decline in their mental health during the pandemic, although overall experienced much higher levels of difficulties compared to their non-EHCP counterparts. There was no evidence of association between ethnicity, SES, and prior physical health with mental health trajectories over this time period although there were differences in average SDQ scores between different ethnic groups and those with different SES status. Paradoxically, CYP with better prior mental health experienced greater worsening during the pandemic; however, these findings need to be interpreted with caution as they could reflect regression to the mean.

The mental health of CYP during the pandemic is somewhat unclear. On the one hand, Samji and colleagues’ [15] systematic review of 116 articles from January 2020 until February 2021 reported an increase in depression and anxiety compared to pre-pandemic estimates, with increased likelihood of poor mental health among older adolescents, girls, and neurodivergent CYP and/or those with chronic physical health conditions. At the same time, a smaller review up to the same period (February 2021) indicates an enormous range of estimates of mental health difficulties such as depression ranging from 6.3% to 71.5% [16]. The systematic review including studies published by February 2022 found little evidence of increased anxiety, mixed findings on depression, conduct and hyperactivity, plus conflicting results for internalising symptoms by respondent [8]. While more recent reports from the Co-SPACE study in the UK indicate that behavioural and attentional difficulties initially increased, they have remained stable from July 2021 to March 2022. They also found that emotional difficulties have increased in the last year but, importantly, this is considered in the context of a population-based trend of increase in such symptoms with age and a convenience sample [6,17]. The findings from this study are also comparable to those from MYRIAD which showed greater deterioration with age for depression, SDQ scores and Warwick Edinburgh Mental Wellbeing Scale scores, and higher SDQ scores predicted by female sex [18].

Our study also points to absolute differences in behavioural, emotional, and attentional difficulties in children with special educational needs and low-income families compared to the rest of the sample [6,17]. Recent data from NHS Digital in the UK also using the SDQ found that rates of a probable mental health disorder in CYP aged 7 to 16 were similar between 2020, 2021, and 2022 and the same was true for young people aged 17 to 19 between 2020 and 2021, but that there was a dramatic increase from 17.4% in 2021 to 25.7% in 2022 [19]. This age group is frequently missed in sampling even though they have consistently reported poorer mental health than school age CYP since 2017 [20] and future research should ensure they are included.

Our findings, when combined with the existing literature, suggest that CYP in their mid to late teens are more vulnerable than younger age groups and continue to struggle with their mental health. There are a number of possible explanations, including impact of pandemic restrictions on social and emotional development during the critically important developmental stage of adolescence and on important social transitions from school or college to work or higher education [20]. Findings also consistently emphasise that female sex is a risk factor for worse mental health, and that those with special educational needs are also more vulnerable. However, it is important to consider that within these data are individual differences and there were substantial between individual variance estimates (a variance of 31 means a plus/minus spread of 5 points on the SDQ which is a big variation). There were some young people who experienced more positive mental health during the pandemic and factors such as poor parental mental health (not measured in the current study) are also critical influences on quality of life [21]. Differences between ethnic groups, SES, and prior physical health are consistent across studies, but our findings add that they also do not influence rates of change over time.

The current study benefits from a clearly defined sample frame, validated measures, and longitudinal follow up, but inevitably also has a number of limitations. There is no objective pre-pandemic information on prior physical and mental health to allow for adjustment, and no prospective pre-pandemic measures, introducing risk of recall bias. All assessment of mental health is via self-report, and the study only included young people from 11 years old upwards so it was not possible to try to replicate the National Survey and Co-SPACE findings about how younger children fared. As described elsewhere, the CLoCk sample itself is limited by low response rate, which might introduce selection bias that could limit generalisability since it may be the case that participants with persisting symptoms were more likely to participate than those who were well [10]. It may also therefore be the case that the trajectories reported would not apply if the sample is not representative but instead only included those with poor mental health. Another limitation is that not all participants completed measures at all 3 time points due to the timing of the assessments and as there was no detailed clinical assessment of special educational needs or neurodevelopmental delay, EHCP was used as a proxy. EHCP indicates a certain level of severity and perseverance, so it is likely that some children in the comparison group would also experience special educational needs or developmental delay. It is possible that this group may have struggled, whereas those with an EHCP would be more visible to educational and social care staff so would potentially have received additional support. For some CYP with EHCPs or poor mental health, the social restrictions and school closures may have temporarily improved their mental health by reducing anxiety. This has potential policy implications for personalising support for CYP with poor mental health/in receipt of an EHCP in future. Using changes in mean scores can obscure important individual differences and clinicians should be aware of extrapolating from this study to individual patients. Furthermore, the study did not assess eating difficulties which, considering the emerging data reporting 60.3% of 17- to 19-year-olds had possible eating problems is an important limitation [19]. Similarly, there could be other factors that are associated with the trajectories that we have not examined. Importantly, the CLoCk study is of non-hospitalised children in England and the findings may not generalise to other countries or young people who experienced hospitalisation due to SARS-CoV-2 infection. Finally, and importantly, as an observational study, this descriptive analysis cannot infer cause and effect. Moreover, without a control group, it is not possible to definitively attribute any of the changes over time to the pandemic but such a control group is clearly impossible. The findings should, however, be viewed in the context of variations in the SDQ under normal circumstances, for example, in which younger boys tend to have more behavioural difficulties than younger girls, but older girls have more emotional difficulties than boys [19,22].

Despite the limitations, this unique study adds to the literature on CYP’s mental health during the pandemic and vulnerability factors that are associated with mental health trajectories, particularly change in mental health trajectories over time. Long-term follow-up of such cohorts is necessary to further understand mental health trajectories as young people emerge from the pandemic.

## Supporting information

S1 STROBE Checklist STROBE Statement—Checklist of items that should be included in reports of cohort studies. *Give information separately for exposed and unexposed groups. **Note:** An Explanation and Elaboration article discusses each checklist item and gives methodological background and published examples of transparent reporting. The STROBE checklist is best used in conjunction with this article (freely available on the Web sites of PLoS Medicine at http://www.plosmedicine.org/, Annals of Internal Medicine at http://www.annals.org/, and Epidemiology at http://www.epidem.com/). Information on the STROBE Initiative is available at http://www.strobe-statement.org.
(DOCX)

S1 Table SDQ subscale scores by data collection schedule: Mean (SD). Note. ^a^*n* = 4,251; ^a^*n* = 7,982; ^c^*n* = 7,328; 3 months from testing reflects the time window: 13 April 2021 to 17 October 2021; 6 months from testing reflects the time window: 13 April 2021 to 17 February 2022; 12 months from testing reflects the time window: 5 October 2021 to 10 May 2022. m = months; SD = standard deviation; SDQ, Strengths and Difficulties Questionnaire.
(DOCX)

S2 Table Demographics of (i) target population*, (ii) participants excluded from the analytical sample, and (iii) participants included in the analytical sample. *Target population includes those who at first invitation 3 or 6 months post-testing filled in their first questionnaire. **p-value from chi-2 test comparing included to excluded participants.
(DOCX)

S3 Table Model coefficients from mixed-effects (random intercept) models: Total SDQ score over time for the whole sample and from models with interactions between time and age, sex, ethnicity, IMD, EHCP status, prior mental health, prior physical health, and baseline PCR status (*N* = 8,518). Note. Separate models were fitted to explore associations between time on total SDQ score (Model 0) and also by age (Model 1), sex (Model 2), ethnicity (Model 3), IMD (Model 4), EHCP (Model 5), prior mental health (Model 6), prior physical health (Model 7), and baseline PCR status (Model 8). Models 0–7 were additionally adjusted for baseline SARS-CoV-2 PCR result. CI = confidence interval; EHCP = educational health and care plan; IMD = Index of Multiple Deprivation; ref = reference category; SDQ = Strengths and Difficulties Questionnaire; SE = standard error.
(DOCX)

S1 File Additional CLoCk Consortium Members. (DOCX)

S1 Fig Average (95% CIs) total difficulties score (Strengths and Difficulties Questionnaire) over time by ethnic group (*N* = 8,518). Footnote: Time was modelled as a continuous variable; time points reflect the mean return time (in months) within each time window. The model was adjusted for SARS-CoV-2 PCR result. The individual-level random effect and residual variance are 32.1 and 9.60, respectively, giving an ICC of 0.77.
(TIF)

S2 Fig Average (95% CIs) total difficulties score (Strengths and Difficulties Questionnaire) over time by Index of Multiple Deprivation (*N* = 8,518). Footnote: Time was modelled as a continuous variable; time points reflect the mean return time (in months) within each time window. The model was adjusted for SARS-CoV-2 PCR result. The individual-level random effect and residual variance are 32.0 and 9.60, respectively, giving an ICC of 0.77.
(TIF)

S3 Fig Average (95% CIs) total difficulties score (Strengths and Difficulties Questionnaire) over time by previous physical health status (*N* = 8,518). Footnote: Time was modelled as a continuous variable; time points reflect the mean return time (in months) within each time window. The model was adjusted for SARS-CoV-2 PCR result. The individual-level random effect and residual variance are 29.7 and 9.60, respectively, giving an ICC of 0.76.
(TIF)

S4 Fig Average (95% CIs) total difficulties score (Strengths and Difficulties Questionnaire) over time by baseline PCR status (*N* = 8,518). Footnote: Time was modelled as a continuous variable; time points reflect the mean return time (in months) within each time window.
(TIF)

## Abbreviations

BIC: Bayesian information criterion
CI: confidence interval
COVID-19: Coronavirus Disease 2019
CYP: children and young people
EHCP: educational health and care plan
IMD: Index of Multiple Deprivation
SARS-CoV-2: Severe Acute Respiratory Syndrome Coronavirus 2
SD: standard deviation
SDQ: Strengths and Difficulties Questionnaire
SE: standard error
SES: socioeconomic status
