# Supplementary material for: Mental health in the COVID-19 pandemic: A longitudinal analysis of the CLoCk cohort study
Source: PLoS Med. 2024 Jan 24;21(1):e1004315. doi: 10.1371/journal.pmed.1004315 (PMC10807843; doi:10.1371/journal.pmed.1004315)
Supplement: S1 Table — Note. an = 4,251; an = 7,982; cn = 7,328; 3 months from testing reflects the time window: 13 April 2021 to 17 October 2021; 6 months from testing reflects the time window: 13 April 2021 to 17 February 2022; 12 months from testing reflects the time window: 5 October 2021 to 10 May 2022. m = months; SD = standard deviation; SDQ, Strengths and Difficulties Questionnaire. (DOCX) [file pmed.1004315.s002.docx]

| Supplementary Table S1. SDQ subscale scores by data collection schedule: Mean (SD) | | | |
| --- | --- | --- | --- |
| Variable | 3m post-testing  (*n* = 4292) | 6m post-testing  (*n* = 8076) |  |
|  |  |  |  |
| Emotional | 3.6 (2.6) | 3.7 (2.6) |  |
|  |  |  |  |
| Conduct | 1.6 (1.6) | 1.6 (1.6) |  |
|  |  |  |  |
| Hyperactivity | 3.9 (2.6) | 3.9 (2.6) |  |
|  |  |  |  |
| Peer relationships | 2.2 (1.9) | 2.2 (1.9) |  |
|  |  |  |  |
| Prosocial skills | 7.6 (1.8) | 7.7 (1.8) |  |
|  |  |  |  |
| Impact | 0.9 (1.8)^a^ | 0.9 (1.8)^b^ |  |
| *Note. ^a^n = 4251; ^a^n = 7982; ^c^n = 7328. 3 months from testing reflects the time window: 13 April 2021 to 17 October 2021; 6 months from testing reflects the time window: 13 April 2021 to 17 February 2022; 12 months from testing reflects the time window: 5 October 2021 to 10 May 2022. m = months; SD = Standard Deviation; Strengths and Difficulties Questionnaire.* | | | |
