# Supplementary material for: Mental health in the COVID-19 pandemic: A longitudinal analysis of the CLoCk cohort study
Source: PLoS Med. 2024 Jan 24;21(1):e1004315. doi: 10.1371/journal.pmed.1004315 (PMC10807843; doi:10.1371/journal.pmed.1004315)
Supplement: S2 Table — *Target population includes those who at first invitation 3 or 6 months post-testing filled in their first questionnaire. **p-value from chi-2 test comparing included to excluded participants. (DOCX) [file pmed.1004315.s003.docx]

**Supplementary Table S2.** Demographics of (i) target population*, (ii) participants excluded from the analytical sample and (iii) participants included in the analytical sample

|  | **Target population***  **(N=17,918)** | **Excluded sample**  **(N=9,400)** | **Included sample**  **(N=8,518)** | **p-value**** |
| --- | --- | --- | --- | --- |
|  |  |  | **Response rate: 47.5%** |  |
| **Sex** |  |  |  | <0.001 |
| Female | 11,132 (62.1) | 5,603 (59.6) | 5,529 (64.9) |  |
| Male | 6,786 (37.9) | 3,797 (40.4) | 2,989 (35.1) |  |
| **Age (years)** |  |  |  | <0.001 |
| 11-14 | 7,776 (43.4) | 4,253 (45.2) | 3,523 (41.4) |  |
| 15-17 | 10,142 (56.6) | 5,147 (54.8) | 4,995 (58.6) |  |
|  |  |  |  |  |
| **Ethnicity** |  |  |  | <0.001 |
| White | 13,532 (75.5) | 6,930 (73.7) | 6,602 (77.5) |  |
| Asian, Asian British | 2,605 (14.5) | 1,463 (15.6) | 1,142 (13.4) |  |
| Mixed | 841 (4.7) | 430 (4.6) | 411 (4.8) |  |
| Black, African, Caribbean | 519 (2.9) | 295 (3.1) | 224 (2.6) |  |
| Other | 304 (1.7) | 203 (2.2) | 101 (1.2) |  |
| Unknown | 117 (0.7) | 79 (0.8) | 38 (0.5) |  |
|  |  |  |  |  |
| **Region** |  |  |  | <0.001 |
| East Midlands | 1,773 (9.9) | 900 (9.6) | 873 (10.3) |  |
| East of England | 2,035 (11.4) | 966 (10.3) | 1,069 (12.6) |  |
| London | 2,358 (13.2) | 1,259 (13.4) | 1,099 (12.9) |  |
| North East England | 990 (5.5) | 567 (6.0) | 423 (5.0) |  |
| North West England | 2,724 (15.2) | 1,510 (16.1) | 1,214 (14.3) |  |
| South East England | 2,448 (13.7) | 1,168 (12.4) | 1,280 (15.0) |  |
| South West England | 1,302 (7.3) | 649 (6.9) | 653 (7.7) |  |
| West Midlands | 2,306 (12.9) | 1,236 (13.2) | 1,070 (12.6) |  |
| Yorkshire and the Humber | 1,982 (11.1) | 1,145 (12.2) | 837 (9.8) |  |
| **IMD quintile*** |  |  |  | <0.001 |
| 1 (most deprived) | 3,732 (20.8) | 2,160 (23.0) | 1,572 (18.5) |  |
| 2 | 3,287 (18.3) | 1,737 (18.5) | 1,550 (18.2) |  |
| 3 | 3,298 (18.4) | 1,722 (18.3) | 1,576 (18.5) |  |
| 4 | 3,634 (20.3) | 1,833 (19.5) | 1,801 (21.1) |  |
| 5 (least deprived) | 3,967 (22.1) | 1,948 (20.7) | 2,019 (23.7) |  |
| **Previous Physical Health** |  |  |  | 0.226 |
| Very good/good | 13,847 (77.3) | 7,216 (76.8) | 6,631 (77.9) |  |
| OK | 3,711 (20.7) | 1,991 (21.2) | 1,720 (20.2) |  |
| Poor/Very poor | 360 (2.0) | 193 (2.1) | 167 (2.0) |  |
| **Previous Mental Health** |  |  |  | 0.527 |
| Very good/good | 1,636 (9.1) | 838 (8.9) | 798 (9.4) |  |
| OK | 5,158 (28.8) | 2,700 (28.7) | 2,458 (28.9) |  |
| Poor/Very poor | 11,124 (62.1) | 5,862 (62.4) | 5,262 (61.8) |  |
| **Educational health and care plan** |  |  |  | 0.422 |
| No | 16,899 (94.3) | 8,853 (94.2) | 8,046 (94.5) |  |
| Yes | 1,019 (5.7) | 547 (5.8) | 472 (5.5) |  |

**target population includes those who at first invitation 3- or 6-months post-testing filled in their first questionnaire.**p-value from chi-2 test comparing included to excluded participants.*
