# Supplementary material for: Mental health in the COVID-19 pandemic: A longitudinal analysis of the CLoCk cohort study
Source: PLoS Med. 2024 Jan 24;21(1):e1004315. doi: 10.1371/journal.pmed.1004315 (PMC10807843; doi:10.1371/journal.pmed.1004315)
Supplement: S3 Table — Note. Separate models were fitted to explore associations between time on total SDQ score (Model 0) and also by age (Model 1), sex (Model 2), ethnicity (Model 3), IMD (Model 4), EHCP (Model 5), prior mental health (Model 6), prior physical health (Model 7), and baseline PCR status (Model 8). Models 0–7 were additionally adjusted for baseline SARS-CoV-2 PCR result. CI = confidence interval; EHCP = educational health and care plan; IMD = Index of Multiple Deprivation; ref = reference category; SDQ = Strengths and Difficulties Questionnaire; SE = standard error. (DOCX) [file pmed.1004315.s004.docx]

| Supplementary Table S3. Model coefficients from mixed-effects (random intercept) models: Total SDQ score over time for the whole sample and from models with interactions between time and age, sex, ethnicity, IMD, EHCP status, prior mental health, prior physical health and baseline PCR status (N = 8,518) | | | | |
| --- | --- | --- | --- | --- |
| Model | **Unstandardised**  ***B* Coefficient** | **SE** | **95% CI** | ***p* value** |
| Model 0  time | 0.11 | 0.01 | 0.09 to 0.12 | <0.001 |
| Constant | 11.16 | 0.10 | 10.97 to 11.34 | <0.001 |
| Model 1  Age [ref.: 11-14 years old] | 1.65 | 0.14 | 1.37 to 1.93 | <0.001 |
| Time | 0.08 | 0.01 | 0.06 to 0.10 | <0.001 |
| Time*age  [ref.: 11-14 years old] | 0.04 | 0.01 | 0.02 to 0.07 | 0.002 |
| constant | 10.17 | 0.13 | 9.92 to 10.42 | <0.001 |
| Model 2  Sex [ref.: girls] | -2.15 | 0.15 | -2.44 to -1.86 | <0.001 |
| Time | 0.12 | 0.01 | 0.10 to 0.13 | <0.001 |
| Time*sex [ref.: girls] | -0.03 | 0.01 | -0.06 to -0.004 | 0.024 |
| constant | 11.9 | 0.11 | 11.7 to 12.1 | <0.001 |
| Model 3  Ethnicity [ref.: Asian/Asian British] |  |  |  | 0.001 |
| Black/African/Caribbean | 0.29 | 0.48 | -0.66 to 1.23 |  |
| Mixed | 1.17 | 0.38 | 0.42 to 1.92 |  |
| Other | 0.17 | 0.69 | -1.18 to 1.52 |  |
| Unknown/prefer not to say | 2.56 | 1.10 | 0.42 to 4.70 |  |
| White | 0.76 | 0.21 | 0.35 to 1.18 |  |
| Time | 0.12 | 0.02 | 0.08 to 0.15 | <0.001 |
| Time*ethnicity  [ref.: Asian/Asian British] |  |  |  | 0.96 |
| Black/African/Caribbean | -0.03 | 0.05 | -0.12 to 0.05 |  |
| Mixed | -0.03 | 0.04 | -0.10 to 0.04 |  |
| Other | -0.00 | 0.06 | -0.13 to 0.12 |  |
| Unknown/prefer not to say | 0.00 | 0.11 | -0.21 to 0.21 |  |
| White | -0.01 | 0.02 | -0.05 to 0.03 |  |
| Constant | 10.5 | 0.21 | 10.1 to 10.9 | <0.001 |
| Model 4  IMD [ref.: IMD 1] |  |  |  | <0.001 |
| IMD 2 | -0.45 | 0.24 | -0.91 to 0.02 |  |
| IMD 3 | -0.67 | 0.24 | -1.14 to -0.21 |  |
| IMD 4 | -1.19 | 0.23 | -1.64 to -0.74 |  |
| IMD 5 | -1.47 | 0.22 | -1.91 to -1.04 |  |
| Time | 0.10 | 0.02 | 0.07 to 0.14 | <0.001 |
| Time*IMD  [ref.: IMD 1] |  |  |  | 0.82 |
| IMD 2 | 0.01 | 0.02 | -0.04 to 0.05 |  |
| IMD 3 | 0.00 | 0.02 | -0.04 to 0.05 |  |
| IMD 4 | -0.01 | 0.02 | -0.06 to 0.03 |  |
| IMD 5 | 0.01 | 0.02 | -0.03 to 0.05 |  |
| Constant | 12.0 | 0.18 | 11.6 to 12.3 | <0.001 |
| Model 5  EHCP  [ref.: no EHCP] | 4.60 | 0.31 | 3.99 to 5.21 | <0.001 |
| Time | 0.11 | 0.01 | 0.10 to 0.12 | <0.001 |
| Time*EHCP  [ref.: no EHCP] | -0.08 | 0.03 | -0.14 to -0.02 | 0.010 |
| Constant | 10.9 | 0.10 | 10.7 to 11.1 | <0.001 |
| Model 6  prior mental health  [ref.: poor/very poor] |  |  |  | <0.001 |
| Okay | -4.78 | 0.24 | -5.25 to -4.32 |  |
| Good/very good | -9.89 | 0.22 | -10.3 to -9.45 |  |
| Time | -0.01 | 0.02 | -0.05 to 0.04 | 0.74 |
| Time*prior mental health  [ref.: poor/very poor] |  |  |  | <0.001 |
| Okay | 0.09 | 0.03 | 0.04 to 0.14 |  |
| Good/very good | 0.14 | 0.02 | 0.10 to 0.19 |  |
| Constant | 18.5 | 0.21 | 18.1 to 18.9 | <0.001 |
| Model 7  prior physical health  [ref.: poor/very poor] |  |  |  | <0.001 |
| Okay | -2.39 | 0.52 | -3.40 to -1.37 |  |
| Good/very good | -6.14 | 0.50 | -7.12 to -5.16 |  |
| Time | 0.03 | 0.05 | -0.06 to 0.12 | 0.48 |
| Time*prior physical health  [ref.: poor/very poor] |  |  |  | 0.10 |
| Okay | 0.04 | 0.05 | -0.06 to 0.14 |  |
| Good/very good | 0.08 | 0.05 | -.01 to 0.18 |  |
| Constant | 16.3 | 0.50 | 15.4 to 17.3 | <0.001 |
| Model 8  PCR status [ref.: negative] | -0.23 | 0.14 | -0.51 to 0.06 | 0.12 |
| Time | 0.11 | 0.01 | 0.09 to 0.13 | <0.01 |
| Time*PCR  [ref.: -ves] | -0.01 | 0.01 | -0.04 to 0.02 | 0.55 |
| constant | 11.1 | 0.10 | 10.9 to 11.3 | <0.001 |
| *Note. Separate models were fitted to explore associations between time on total SDQ score (Model 0) and also by age (Model 1), sex (Model 2), ethnicity (Model 3), IMD (Model 4), EHCP (Model 5), prior mental health (Model 6), prior physical health (Model 7) and baseline PCR status (Model 8). Models0-7 were additionally adjusted for baseline SARS-CoV-2 PCR result. CI = Confidence Interval; EHCP = Educational Health and Care Plan; IMD = Index of Multiple Deprivation; ref = reference category; SDQ = Strengths and Difficulties Questionnaire; SE = Standard Error.* | | | | |
