# Supplementary material for: Mental health in the COVID-19 pandemic: A longitudinal analysis of the CLoCk cohort study
Source: PLoS Med. 2024 Jan 24;21(1):e1004315. doi: 10.1371/journal.pmed.1004315 (PMC10807843; doi:10.1371/journal.pmed.1004315)
Supplement: S1 File — (DOCX) [file pmed.1004315.s005.docx]

Additional CLoCk Consortium Members

| **Name** | **Institution** | **Email** |
| --- | --- | --- |
| Marta Buszewicz | University College London | [m.buszewicz@ucl.ac.uk](about:blank) |
| Esther Crawley | University of Bristol | [Esther.Crawley@bristol.ac.uk](about:blank) |
| Bianca De Stavola | University College London | [b.destavola@ucl.ac.uk](about:blank) |
| Shruti Garg | University of Manchester | [Shruti.Garg@mft.nhs.uk](about:blank) |
| Anthony Harnden | Oxford University | [anthony.harnden@phc.ox.ac.uk](about:blank) |
| Michael Levin | Imperial College London | [m.levin@imperial.ac.uk](about:blank) |
| Vanessa Poustie | University of Liverpool | [v.poustie@liverpool.ac.uk](about:blank) |
| Terry Segal | University College London Hospitals NHS Foundation Trust | [terry.segal@nhs.net](about:blank) |
| Malcolm Semple | University of Liverpool | [M.G.Semple@liverpool.ac.uk](about:blank) |
| Shamez Ladhani | St. George’s University of London and UK Health Security Agency | [shamez.ladhani@ukhsa.gov.uk](about:blank) |
| Elizabeth Whittaker | Imperial College London | [e.whittaker@imperial.ac.uk](about:blank) |
