# Supplementary figures and images for: Mental health in the COVID-19 pandemic: A longitudinal analysis of the CLoCk cohort study
Source: PLoS Med. 2024 Jan 24;21(1):e1004315. doi: 10.1371/journal.pmed.1004315 (PMC10807843; doi:10.1371/journal.pmed.1004315)

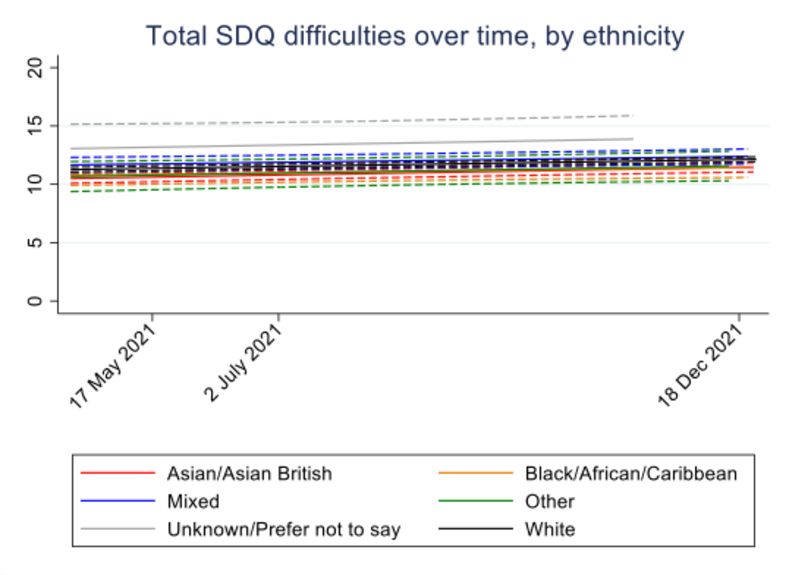

Supplement: S1 Fig — Footnote: Time was modelled as a continuous variable; time points reflect the mean return time (in months) within each time window. The model was adjusted for SARS-CoV-2 PCR result. The individual-level random effect and residual variance are 32.1 and 9.60, respectively, giving an ICC of 0.77. (TIF) [file pmed.1004315.s006.tif]

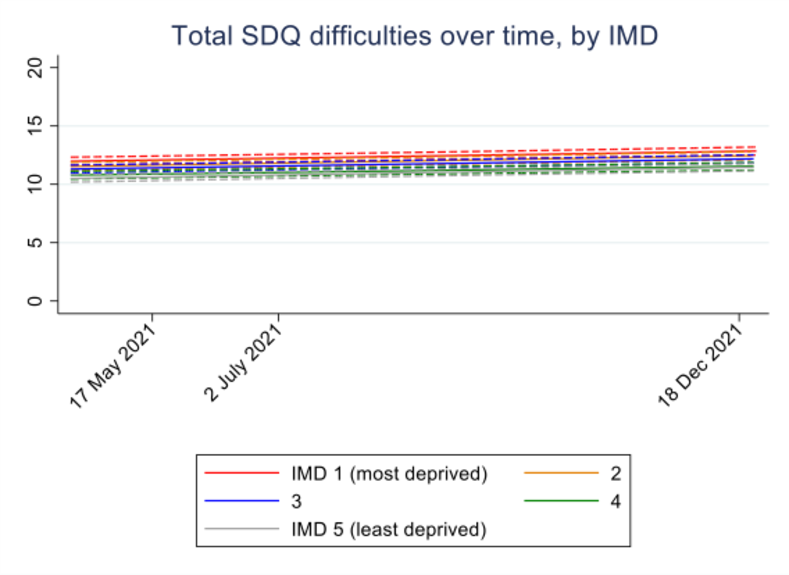

Supplement: S2 Fig — Footnote: Time was modelled as a continuous variable; time points reflect the mean return time (in months) within each time window. The model was adjusted for SARS-CoV-2 PCR result. The individual-level random effect and residual variance are 32.0 and 9.60, respectively, giving an ICC of 0.77. (TIF) [file pmed.1004315.s007.tif]

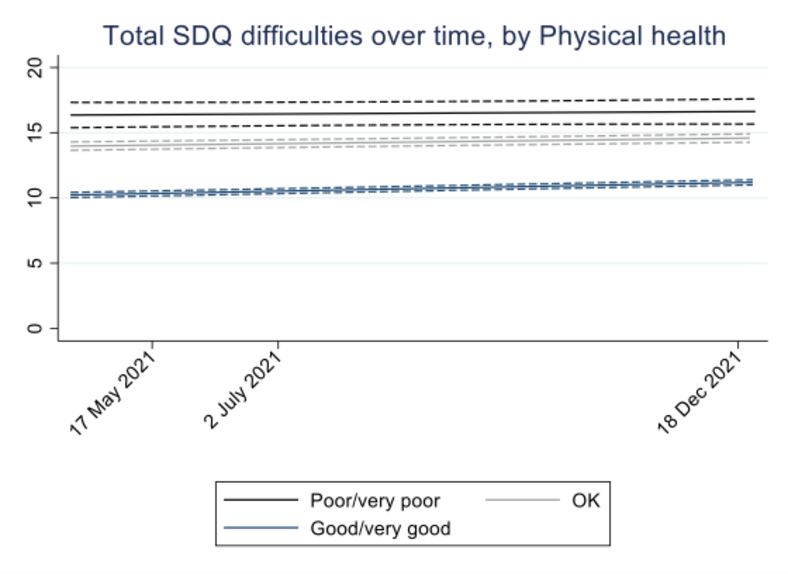

Supplement: S3 Fig — Footnote: Time was modelled as a continuous variable; time points reflect the mean return time (in months) within each time window. The model was adjusted for SARS-CoV-2 PCR result. The individual-level random effect and residual variance are 29.7 and 9.60, respectively, giving an ICC of 0.76. (TIF) [file pmed.1004315.s008.tif]

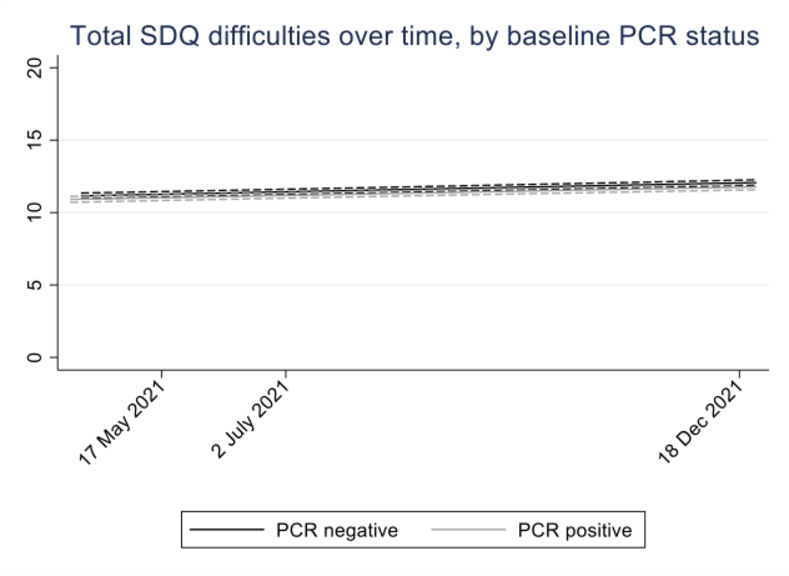

Supplement: S4 Fig — Footnote: Time was modelled as a continuous variable; time points reflect the mean return time (in months) within each time window. (TIF) [file pmed.1004315.s009.tif]
